# 1,4-Dibenzyl­piperazine

**DOI:** 10.1107/S1600536810049111

**Published:** 2010-11-27

**Authors:** Meng Zhang, Yong-hong Zhou, Li-hong Hu, Xiao-hui Yang

**Affiliations:** a Institute of Chemical Industry of Forest Products, Chinese Academy of Forestry, Nanjing 210042, People’s Republic of China, and Jiangsu Qiang Lin Bio-Energy Co. Ltd, Liyang 213364, People’s Republic of China

## Abstract

In the title compound, C_18_H_22_N_2_, which possesses non-crystallographic inversion symmetry, the central piperazine ring adopts a chair conformation. The phenyl rings are not exactly parallel and make a dihedral angle of 1.3 (1)°. No significant inter­molecular contacts are observed in the crystal.

## Related literature

For the properties and applications of piperazine derivatives, see: Zhao *et al.* (2002 ▶); Sonurlikar *et al.* (1977 ▶); Bigoli *et al.* (2001 ▶). For the synthesis of related compounds, see: Zheng *et al.* (2005 ▶); Sarangarajan *et al.* (2005 ▶). For related structures, see: Yogavel *et al.* (2003 ▶); Gunasekaran *et al.* (1996 ▶); Thiru­murugan *et al.* (1998 ▶).
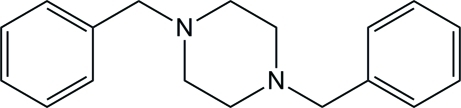


## Experimental

### 

#### Crystal data

C_18_H_22_N_2_
*M* *_r_* = 266.38
Orthorhombic, 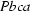

*a* = 7.5130 (15) Å
*b* = 19.127 (4) Å
*c* = 21.366 (4) Å
*V* = 3070.3 (11) Å^3^
*Z* = 8
Mo *K*α radiation
μ = 0.07 mm^−1^
*T* = 293 K
0.30 × 0.20 × 0.10 mm

#### Data collection

Enraf–Nonius CAD-4 diffractometer
Absorption correction: ψ scan (North *et al.*, 1968 ▶) *T*_min_ = 0.980, *T*_max_ = 0.993
5468 measured reflections
2781 independent reflections
1650 reflections with *I* > 2σ(*I*)
*R*_int_ = 0.045
3 standard reflections every 200 reflections  intensity decay: 1%

#### Refinement

*R*[*F* ^2^ > 2σ(*F* ^2^)] = 0.052
*wR*(*F* ^2^) = 0.141
*S* = 1.01
2781 reflections
182 parameters
H-atom parameters constrained
Δρ_max_ = 0.16 e Å^−3^
Δρ_min_ = −0.13 e Å^−3^

### 

Data collection: *CAD-4 EXPRESS* (Enraf–Nonius, 1989 ▶); cell refinement: *CAD-4 EXPRESS*; data reduction: *XCAD4* (Harms & Wocadlo, 1995 ▶); program(s) used to solve structure: *SHELXS97* (Sheldrick, 2008 ▶); program(s) used to refine structure: *SHELXL97* (Sheldrick, 2008 ▶); molecular graphics: *SHELXTL* (Sheldrick, 2008 ▶); software used to prepare material for publication: *SHELXTL*.

## Supplementary Material

Crystal structure: contains datablocks I, global. DOI: 10.1107/S1600536810049111/bh2322sup1.cif

Structure factors: contains datablocks I. DOI: 10.1107/S1600536810049111/bh2322Isup2.hkl

Additional supplementary materials:  crystallographic information; 3D view; checkCIF report

## Figures and Tables

**Fig. 1.**
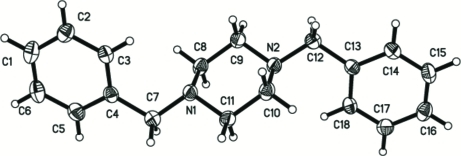
A view of the molecular structure of the title compound, showing displacement ellipsoids at the 30% probability level. Hydrogen atoms are drawn as spheres of arbitrary radius.

**Fig. 2.**
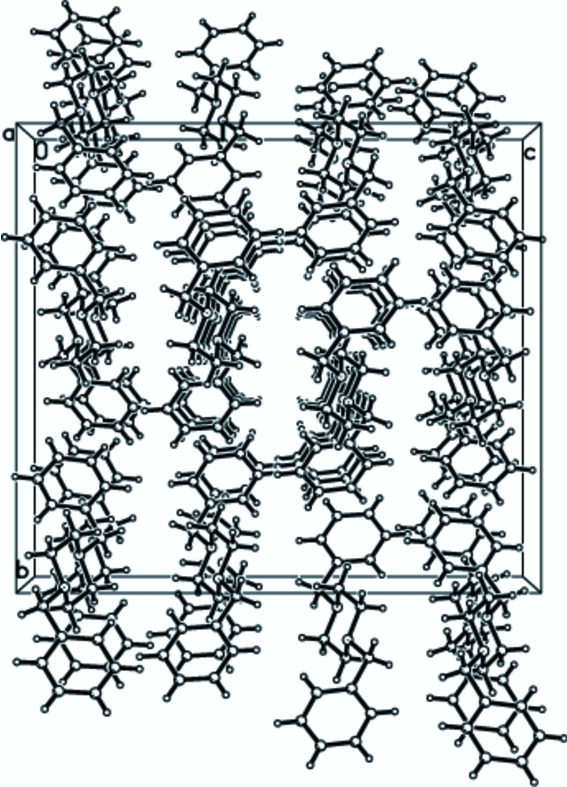
The packing of the title compound, viewed along the a axis.

## supplementary crystallographic information

### Comment

The structural study of piperazine derivatives is of interest, because some piperazine-containing derivatives constitute a novel class of mixed D2/D4 receptor antagonists (Zhao *et al.*, 2002), and disubstituted piperazine derivatives are antifilarial, antiamoebic and spermicidal agents (Sonurlikar *et al.*, 1977). In addition, piperazine derivatives are useful precursors of mixed-ligand dithiolenes of interest for non-linear optics (Bigoli *et al.*, 2001). Recently, many piperazine derivatives with various substituents have been synthesized (Zheng *et al.*, 2005; Sarangarajan *et al.*, 2005). Herein, we report the crystal structure of the title compound, (I).

The geometry and labeling scheme of the title compound are depicted in Fig. 1, and the packing structure is given in Fig. 2. The piperazine ring exhibits a chair conformation with the usual bond lengths and angles (Yogavel *et al.*, 2003), comparable with those of related reported structures (Gunasekaran *et al.*, 1996; Thirumurugan *et al.*, 1998).

### Experimental

To a solution of anhydrous piperazine (5 mmol, 0.43 g) in CH_2_Cl_2_ (20 ml) was added 2.2 equivalents of triethylamine (1.5 ml), followed by benzyl bromide (10 mmol, 2.66 g) in CH_2_Cl_2_ (20 ml). After the mixture had been stirred for 10 min., the solvent was removed using a rotary evaporator. The solid residue was washed with water and recrystallized from ethanol-cyclohexane to give a colourless solid (76% yield). Crystals of (I) suitable for X-ray diffraction were obtained by slow evaporation of an ethanol solution.

### Refinement

All H atoms were placed in geometrically idealized positions and constrained to ride on their parent atoms, with C—H distances in the range 0.93–0.97 Å, and *U*_iso_(H) = 1.2 *U*_eq_ of the carrier atom.

### Crystal data

**Table:**

C_18_H_22_N_2_	*D*_x_ = 1.153 Mg m^−^^3^
*M**_r_* = 266.38	Melting point: 372 K
Orthorhombic, *P**b**c**a*	Mo *K*α radiation, λ = 0.71073 Å
Hall symbol: -P 2ac 2ab	Cell parameters from 25 reflections
*a* = 7.5130 (15) Å	θ = 9–13°
*b* = 19.127 (4) Å	µ = 0.07 mm^−^^1^
*c* = 21.366 (4) Å	*T* = 293 K
*V* = 3070.3 (11) Å^3^	Strip, colorless
*Z* = 8	0.30 × 0.20 × 0.10 mm
*F*(000) = 1152	

### Data collection

**Table:**

Enraf–Nonius CAD-4 diffractometer	1650 reflections with *I* > 2σ(*I*)
Radiation source: fine-focus sealed tube	*R*_int_ = 0.045
graphite	θ_max_ = 25.3°, θ_min_ = 1.9°
ω/2θ scans	*h* = 0→9
Absorption correction: ψ scan (North *et al.*, 1968)	*k* = 0→22
*T*_min_ = 0.980, *T*_max_ = 0.993	*l* = −25→25
5468 measured reflections	3 standard reflections every 200 reflections
2781 independent reflections	intensity decay: 1%

### Refinement

**Table:**

Refinement on *F*^2^	Secondary atom site location: difference Fourier map
Least-squares matrix: full	Hydrogen site location: inferred from neighbouring sites
*R*[*F*^2^ > 2σ(*F*^2^)] = 0.052	H-atom parameters constrained
*wR*(*F*^2^) = 0.141	*w* = 1/[σ^2^(*F*_o_^2^) + (0.065*P*)^2^] where *P* = (*F*_o_^2^ + 2*F*_c_^2^)/3
*S* = 1.00	(Δ/σ)_max_ < 0.001
2781 reflections	Δρ_max_ = 0.16 e Å^−^^3^
182 parameters	Δρ_min_ = −0.13 e Å^−^^3^
0 restraints	Extinction correction: *SHELXL97* (Sheldrick, 2008), Fc^*^=kFc[1+0.001xFc^2^λ^3^/sin(2θ)]^-1/4^
0 constraints	Extinction coefficient: 0.0097 (11)
Primary atom site location: structure-invariant direct methods	

### Fractional atomic coordinates and isotropic or equivalent isotropic displacement parameters (Å^2^)

**Table:**

	*x*	*y*	*z*	*U*_iso_*/*U*_eq_	
N1	0.1744 (2)	0.09152 (8)	0.62991 (8)	0.0427 (5)	
C1	0.6103 (3)	0.28645 (12)	0.57631 (12)	0.0604 (7)	
H1A	0.6752	0.3217	0.5569	0.073*	
N2	−0.1771 (2)	0.03706 (8)	0.61285 (8)	0.0432 (5)	
C2	0.5050 (3)	0.24229 (12)	0.54133 (10)	0.0533 (6)	
H2A	0.4989	0.2477	0.4981	0.064*	
C3	0.4087 (3)	0.19002 (11)	0.57034 (10)	0.0449 (6)	
H3A	0.3382	0.1604	0.5463	0.054*	
C4	0.4150 (3)	0.18090 (10)	0.63433 (10)	0.0413 (5)	
C5	0.5204 (3)	0.22622 (11)	0.66895 (11)	0.0542 (6)	
H5A	0.5252	0.2218	0.7123	0.065*	
C6	0.6185 (3)	0.27799 (12)	0.63953 (13)	0.0634 (7)	
H6A	0.6908	0.3073	0.6632	0.076*	
C7	0.3183 (3)	0.12199 (11)	0.66679 (10)	0.0504 (6)	
H7A	0.4034	0.0855	0.6769	0.061*	
H7B	0.2696	0.1393	0.7059	0.061*	
C8	0.0184 (3)	0.13693 (10)	0.62714 (10)	0.0474 (6)	
H8A	0.0520	0.1819	0.6098	0.057*	
H8B	−0.0268	0.1446	0.6691	0.057*	
C9	−0.1255 (3)	0.10464 (10)	0.58725 (10)	0.0480 (6)	
H9A	−0.2282	0.1354	0.5860	0.058*	
H9B	−0.0824	0.0987	0.5448	0.058*	
C10	−0.0218 (3)	−0.00859 (10)	0.61446 (10)	0.0483 (6)	
H10A	0.0230	−0.0154	0.5723	0.058*	
H10B	−0.0551	−0.0539	0.6313	0.058*	
C11	0.1210 (3)	0.02366 (10)	0.65474 (10)	0.0478 (6)	
H11A	0.0768	0.0293	0.6971	0.057*	
H11B	0.2235	−0.0072	0.6562	0.057*	
C12	−0.3259 (3)	0.00581 (11)	0.57907 (10)	0.0497 (6)	
H12A	−0.2825	−0.0132	0.5399	0.060*	
H12B	−0.4116	0.0421	0.5692	0.060*	
C13	−0.4189 (3)	−0.05156 (10)	0.61511 (9)	0.0396 (5)	
C14	−0.5306 (3)	−0.09799 (11)	0.58415 (10)	0.0494 (6)	
H14A	−0.5413	−0.0954	0.5408	0.059*	
C15	−0.6260 (3)	−0.14791 (12)	0.61664 (12)	0.0569 (6)	
H15A	−0.7012	−0.1782	0.5952	0.068*	
C16	−0.6102 (3)	−0.15299 (11)	0.68032 (12)	0.0555 (6)	
H16A	−0.6745	−0.1866	0.7022	0.067*	
C17	−0.4989 (3)	−0.10828 (11)	0.71169 (10)	0.0505 (6)	
H17A	−0.4870	−0.1119	0.7549	0.061*	
C18	−0.4041 (3)	−0.05765 (11)	0.67923 (10)	0.0440 (6)	
H18A	−0.3295	−0.0274	0.7010	0.053*	

### Atomic displacement parameters (Å^2^)

**Table:**

	*U*^11^	*U*^22^	*U*^33^	*U*^12^	*U*^13^	*U*^23^
N1	0.0338 (10)	0.0385 (10)	0.0558 (11)	−0.0001 (8)	−0.0015 (8)	0.0068 (8)
C1	0.0516 (15)	0.0518 (15)	0.0779 (18)	−0.0086 (12)	0.0179 (14)	−0.0005 (14)
N2	0.0328 (9)	0.0414 (10)	0.0553 (11)	0.0003 (9)	−0.0012 (9)	0.0073 (9)
C2	0.0508 (14)	0.0543 (14)	0.0547 (14)	−0.0011 (13)	0.0103 (12)	0.0035 (11)
C3	0.0366 (12)	0.0465 (13)	0.0516 (14)	−0.0022 (10)	−0.0013 (10)	−0.0029 (11)
C4	0.0316 (11)	0.0426 (12)	0.0496 (13)	0.0028 (10)	−0.0007 (10)	−0.0019 (10)
C5	0.0519 (15)	0.0588 (15)	0.0520 (14)	−0.0035 (13)	−0.0045 (12)	−0.0074 (11)
C6	0.0495 (15)	0.0580 (16)	0.0826 (19)	−0.0142 (13)	−0.0008 (13)	−0.0161 (14)
C7	0.0457 (13)	0.0516 (13)	0.0541 (13)	−0.0050 (11)	−0.0061 (12)	0.0070 (11)
C8	0.0430 (13)	0.0377 (11)	0.0614 (14)	0.0003 (11)	0.0046 (11)	0.0055 (11)
C9	0.0363 (13)	0.0431 (13)	0.0645 (14)	0.0021 (10)	−0.0021 (11)	0.0114 (11)
C10	0.0402 (13)	0.0389 (12)	0.0658 (15)	0.0006 (10)	0.0027 (11)	0.0046 (11)
C11	0.0382 (12)	0.0422 (13)	0.0629 (14)	0.0030 (10)	−0.0014 (11)	0.0118 (11)
C12	0.0423 (13)	0.0561 (14)	0.0508 (13)	−0.0035 (11)	−0.0036 (11)	0.0066 (11)
C13	0.0308 (11)	0.0449 (12)	0.0432 (12)	0.0024 (10)	−0.0008 (10)	−0.0015 (10)
C14	0.0494 (14)	0.0521 (14)	0.0468 (12)	−0.0022 (12)	−0.0062 (11)	−0.0047 (11)
C15	0.0457 (14)	0.0484 (14)	0.0768 (17)	−0.0097 (12)	−0.0082 (12)	−0.0078 (13)
C16	0.0444 (14)	0.0506 (14)	0.0715 (17)	−0.0052 (12)	0.0107 (12)	0.0068 (12)
C17	0.0425 (14)	0.0584 (14)	0.0505 (13)	0.0004 (12)	0.0039 (11)	0.0055 (11)
C18	0.0345 (12)	0.0480 (13)	0.0495 (13)	−0.0042 (10)	0.0009 (10)	−0.0045 (10)

### Geometric parameters (Å, °)

**Table:**

N1—C11	1.458 (2)	C8—H8B	0.9700
N1—C7	1.459 (3)	C9—H9A	0.9700
N1—C8	1.460 (3)	C9—H9B	0.9700
C1—C6	1.362 (3)	C10—C11	1.508 (3)
C1—C2	1.377 (3)	C10—H10A	0.9700
C1—H1A	0.9300	C10—H10B	0.9700
N2—C9	1.456 (2)	C11—H11A	0.9700
N2—C10	1.457 (3)	C11—H11B	0.9700
N2—C12	1.459 (3)	C12—C13	1.512 (3)
C2—C3	1.381 (3)	C12—H12A	0.9700
C2—H2A	0.9300	C12—H12B	0.9700
C3—C4	1.379 (3)	C13—C18	1.379 (3)
C3—H3A	0.9300	C13—C14	1.389 (3)
C4—C5	1.388 (3)	C14—C15	1.381 (3)
C4—C7	1.509 (3)	C14—H14A	0.9300
C5—C6	1.385 (3)	C15—C16	1.369 (3)
C5—H5A	0.9300	C15—H15A	0.9300
C6—H6A	0.9300	C16—C17	1.371 (3)
C7—H7A	0.9700	C16—H16A	0.9300
C7—H7B	0.9700	C17—C18	1.388 (3)
C8—C9	1.509 (3)	C17—H17A	0.9300
C8—H8A	0.9700	C18—H18A	0.9300
			
C11—N1—C7	111.26 (16)	N2—C9—H9B	109.7
C11—N1—C8	108.88 (16)	C8—C9—H9B	109.7
C7—N1—C8	112.29 (16)	H9A—C9—H9B	108.2
C6—C1—C2	119.4 (2)	N2—C10—C11	109.76 (17)
C6—C1—H1A	120.3	N2—C10—H10A	109.7
C2—C1—H1A	120.3	C11—C10—H10A	109.7
C9—N2—C10	109.14 (16)	N2—C10—H10B	109.7
C9—N2—C12	112.43 (16)	C11—C10—H10B	109.7
C10—N2—C12	112.31 (16)	H10A—C10—H10B	108.2
C1—C2—C3	120.1 (2)	N1—C11—C10	110.62 (17)
C1—C2—H2A	120.0	N1—C11—H11A	109.5
C3—C2—H2A	120.0	C10—C11—H11A	109.5
C4—C3—C2	121.2 (2)	N1—C11—H11B	109.5
C4—C3—H3A	119.4	C10—C11—H11B	109.5
C2—C3—H3A	119.4	H11A—C11—H11B	108.1
C3—C4—C5	118.0 (2)	N2—C12—C13	113.54 (17)
C3—C4—C7	122.24 (19)	N2—C12—H12A	108.9
C5—C4—C7	119.7 (2)	C13—C12—H12A	108.9
C6—C5—C4	120.6 (2)	N2—C12—H12B	108.9
C6—C5—H5A	119.7	C13—C12—H12B	108.9
C4—C5—H5A	119.7	H12A—C12—H12B	107.7
C1—C6—C5	120.7 (2)	C18—C13—C14	117.88 (19)
C1—C6—H6A	119.6	C18—C13—C12	122.00 (18)
C5—C6—H6A	119.6	C14—C13—C12	120.04 (19)
N1—C7—C4	113.99 (17)	C15—C14—C13	121.1 (2)
N1—C7—H7A	108.8	C15—C14—H14A	119.5
C4—C7—H7A	108.8	C13—C14—H14A	119.5
N1—C7—H7B	108.8	C16—C15—C14	120.2 (2)
C4—C7—H7B	108.8	C16—C15—H15A	119.9
H7A—C7—H7B	107.6	C14—C15—H15A	119.9
N1—C8—C9	110.77 (16)	C15—C16—C17	119.6 (2)
N1—C8—H8A	109.5	C15—C16—H16A	120.2
C9—C8—H8A	109.5	C17—C16—H16A	120.2
N1—C8—H8B	109.5	C16—C17—C18	120.3 (2)
C9—C8—H8B	109.5	C16—C17—H17A	119.9
H8A—C8—H8B	108.1	C18—C17—H17A	119.9
N2—C9—C8	109.98 (17)	C13—C18—C17	120.9 (2)
N2—C9—H9A	109.7	C13—C18—H18A	119.5
C8—C9—H9A	109.7	C17—C18—H18A	119.5
			
C6—C1—C2—C3	0.1 (3)	C9—N2—C10—C11	59.7 (2)
C1—C2—C3—C4	0.1 (3)	C12—N2—C10—C11	−174.95 (16)
C2—C3—C4—C5	0.4 (3)	C7—N1—C11—C10	−177.43 (17)
C2—C3—C4—C7	−176.9 (2)	C8—N1—C11—C10	58.3 (2)
C3—C4—C5—C6	−1.2 (3)	N2—C10—C11—N1	−60.0 (2)
C7—C4—C5—C6	176.1 (2)	C9—N2—C12—C13	−162.08 (17)
C2—C1—C6—C5	−0.9 (4)	C10—N2—C12—C13	74.4 (2)
C4—C5—C6—C1	1.5 (4)	N2—C12—C13—C18	20.0 (3)
C11—N1—C7—C4	163.51 (17)	N2—C12—C13—C14	−163.34 (18)
C8—N1—C7—C4	−74.2 (2)	C18—C13—C14—C15	1.0 (3)
C3—C4—C7—N1	−19.8 (3)	C12—C13—C14—C15	−175.73 (19)
C5—C4—C7—N1	162.96 (18)	C13—C14—C15—C16	−0.8 (3)
C11—N1—C8—C9	−57.9 (2)	C14—C15—C16—C17	−0.1 (3)
C7—N1—C8—C9	178.46 (16)	C15—C16—C17—C18	0.6 (3)
C10—N2—C9—C8	−59.3 (2)	C14—C13—C18—C17	−0.5 (3)
C12—N2—C9—C8	175.41 (17)	C12—C13—C18—C17	176.23 (19)
N1—C8—C9—N2	59.1 (2)	C16—C17—C18—C13	−0.4 (3)
